# First-in-Human Randomized Study to Assess the Safety and Immunogenicity of an Investigational Respiratory Syncytial Virus (RSV) Vaccine Based on Chimpanzee-Adenovirus-155 Viral Vector–Expressing RSV Fusion, Nucleocapsid, and Antitermination Viral Proteins in Healthy Adults

**DOI:** 10.1093/cid/ciz653

**Published:** 2019-07-24

**Authors:** Paola Cicconi, Claire Jones, Esha Sarkar, Laura Silva-Reyes, Paul Klenerman, Catherine de Lara, Claire Hutchings, Philippe Moris, Michel Janssens, Laurence A Fissette, Marta Picciolato, Amanda Leach, Antonio Gonzalez-Lopez, Ilse Dieussaert, Matthew D Snape

**Affiliations:** 1 Oxford Vaccine Group, Department of Paediatrics, University of Oxford, United Kingdom; 2 Nuffield Department of Medicine, University of Oxford, United Kingdom; 3 GSK, Rixensart, Belgium; 4 GSK, Wavre, Belgium; 5 GSK, Rockville, Maryland; 6 National Institute for Health Research Oxford Biomedical Centre, United Kingdom

**Keywords:** RSV, viral vector vaccine, first-in-human study

## Abstract

**Background:**

Respiratory syncytial virus (RSV) disease is a major cause of infant morbidity and mortality. This Phase I, randomized, observer-blind, placebo- and active-controlled study evaluated an investigational vaccine against RSV (ChAd155-RSV) using the viral vector chimpanzee-adenovirus-155, encoding RSV fusion (F), nucleocapsid, and transcription antitermination proteins.

**Methods:**

Healthy 18–45-year-old adults received ChAd155-RSV, a placebo, or an active control (Bexsero) at Days (D) 0 and 30. An escalation from a low dose (5 × 10^9^ viral particles) to a high dose (5 × 10^10^ viral particles) occurred after the first 16 participants. Endpoints were solicited/unsolicited and serious adverse events (SAEs), biochemical/hematological parameters, cell-mediated immunogenicity by enzyme-linked immunospot, functional neutralizing antibodies, anti RSV-F immunoglobin (Ig) G, and ChAd155 neutralizing antibodies.

**Results:**

There were 7 participants who received the ChAd155-RSV low dose, 31 who received the ChAd155-RSV high dose, 19 who received the placebo, and 15 who received the active control. No dose-related toxicity or attributable SAEs at the 1-year follow-up were observed. The RSV-A neutralizing antibodies geometric mean titer ratios (post/pre-immunization) following a high dose were 2.6 (D30) and 2.3 (D60). The ratio of the fold-rise (D0 to D30) in anti-F IgG over the fold-rise in RSV-A–neutralizing antibodies was 1.01. At D7 after the high dose of the study vaccine, the median frequencies of circulating B-cells secreting anti-F antibodies were 133.3/10^6^ (IgG) and 16.7/10^6^ (IgA) in peripheral blood mononuclear cells (PBMCs). The median frequency of RSV-F–specific interferon γ–secreting T-cells after a ChAd155-RSV high dose was 108.3/10^6^ PBMCs at D30, with no increase after the second dose.

**Conclusions:**

In adults previously naturally exposed to RSV, ChAd155-RSV generated increases in specific humoral and cellular immune responses without raising significant safety concerns.

**Clinical Trials Registration:**

NCT02491463.

## Teaser

We investigated the safety and immunogenicity of a novel chimpanzee-adenovirus-155 vaccine against respiratory syncytial virus (RSV; ChAd155-RSV). In healthy adults with previous natural RSV exposure, ChAd155-RSV generated antigen-specific humoral and cellular immune responses without raising significant safety concerns.

**(See the Editorial Commentary by Korsten and Bont on pages 2082–3.)**

The global incidence of respiratory syncytial virus (RSV)-associated acute lower respiratory tract infections in 2015 was estimated at 33 million cases in children younger than 5 years, with 10% requiring hospital admission and with up to 59 600 deaths, most of which occurred in developing countries [1]. By the age of 3 years, over 85% to 100% of children have been infected. Furthermore, infection with RSV does not convey persistent immunity and reinfection is common [2, 3].

Whereas the severity of infection tends to decrease with age, there is evidence that RSV respiratory infections in early childhood could be associated with long-term wheezing and asthma [4]. The health and economic costs due to RSV infections keeps RSV a priority target for vaccine development.

While the induction of effective antibodies against RSV is a fundamental mechanism to confer protection in the host [5, 6], the incomplete comprehension of the immune mechanism associated with RSV infection remains an obstacle for the development of a safe and effective vaccine. In the late 1960s, a formalin-inactivated whole virus RSV vaccine tested in clinical trials led to enhanced respiratory disease (ERD) in infants, causing a predisposition to an exacerbated immunopathology attributable to an induction of low-quality, non-neutralizing antibodies and a strongly biased Th2-like immune response on exposure to a natural infection [7–9]. This experience has led to heightened safety concerns with pediatric RSV vaccine candidates.

A vaccine based on recombinant adenoviral vectors carrying relevant RSV antigens, mobilizing both the humoral and cellular arms of the immune response, is considered a potential solution to induce a balanced and effective immune response against RSV virus in a naive population [10]. Chimpanzee-derived adenoviruses are good potential vector candidates for multiple infectious agents, as they are well tolerated in human clinical trials, are weakly neutralized by human sera, and have potent immunogenicity, mainly Th1-directed [11–15].

Accordingly, a new RSV vaccine is being developed for pediatric use, based on 3 RSV viral proteins encoded by chimpanzee-derived adenovector 155 (ChAd155-RSV). This investigational vaccine contains the fusion (F) protein, which is a major surface antigen and the main target of the neutralizing antibody (NAb) response [16], and the nucleocapsid (N) and antitermination (M2-1) internal proteins, both known to be sources of many T cell epitopes, for the induction of cell-mediated immunity [17].

The purpose of this first-in-human clinical trial in healthy adults was to evaluate the safety, reactogenicity, and immunogenicity of ChAd155-RSV.

## MATERIALS AND METHODS

### Ethics Statement

The study (ClinicalTrials.gov, NCT02491463) was approved by National Health Service Research Ethics Committee South Central, Berkshire, United Kingdom (reference number 15/SC/0133). A full ethics statement is available in the Supplementary Methods.

### Study Vaccines and Administration

The investigational ChAd155-RSV vaccine is a replicant-deficient recombinant viral vector manufactured using a synthetic DNA fragment from multiple strains encoding for protein F, with deletions from the transmembrane and the cytoplasmic regions (F0ΔTM), N, and M2-1. The vaccination regimen was 2 intramuscular injections of 5 x 10^9^ (low dose [LD]; ChAd155-RSV-LD group) or 5 × 10^10^ (high dose [HD]; ChAd155-RSV-HD group) viral particles in the deltoid of the nondominant arm, according to a 0, 1-month schedule (see Supplementary Methods for the determination of viral particle concentration).

A placebo (saline) group was included as a control for the reactogenicity, safety, and immunogenicity assessments. The use of a group B meningococcal vaccine, 4CMenB (Bexsero, GSK), was included as an active control. This was administered at the same 0, 1-month schedule, allowing the maintenance of blinding throughout the trial.

### Study Design

The primary objective was to evaluate the safety and reactogenicity of 2 doses of ChAd155-RSV, administered in a 0, 1-month schedule, up to 30 days after Dose 2. Secondary objectives were to evaluate the cell-mediated immunogenicity of ChAd155-RSV up to 30 days after Dose 2 and safety up to the study conclusion (Day [D] 360). Tertiary objectives included an assessment of functional antibody titers and evaluation of adenoviral presence in blood up to 30 days after Dose 2, the occurrence of RSV infections up to D360, and further exploratory immunology to detect vaccine-related immune responses, such as specific CD4/CD8 T cell determinations by intracellular cytokine staining/fluorescence-activated cell sorting (ICS/FACS) or antivector immunity.

**Figure 1.**
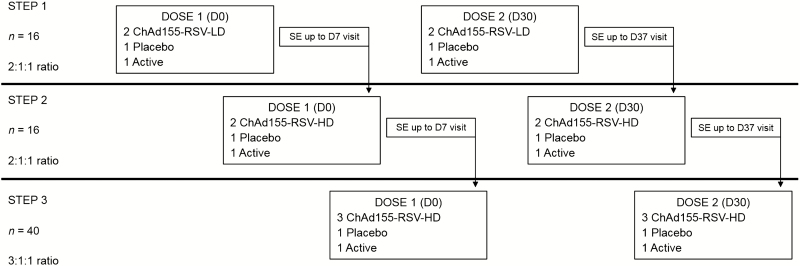
Overview of staggered design and safety evaluation. SE up to D7/D37: safety evaluation by an Internal Safety Review Committee based on all available safety data, and at least safety data up to 7 days after vaccination (including hematology/biochemistry parameters). The placebo was a saline solution and the active dose was an active control. Abbreviations: ChAd155, chimpanzee-adenovirus-155 vaccine; D, day; HD, high dose; LD, low dose; RSV, respiratory syncytial virus; SE, standard error.

This was a Phase I, randomized, placebo- and active-controlled study with 3 parallel groups in a 3-step staggered design (Figure 1). Details of randomization and progression through each step of the study are outlined in the Supplementary Methods. Given the different appearances and storage conditions of the investigational RSV vaccine, placebo, and active control, double blinding was not possible; therefore, administrators of the vaccine were not blinded, whilst participants, staff undertaking clinical evaluations, and laboratory staff were blinded (observer blind). Participants were recruited at the Oxford Vaccine Group, University of Oxford, United Kingdom, between July 2015 and January 2016.

### Safety Assessment

Study visits for safety evaluations occurred on D0 and D30, with 9 additional visits up to D360. Safety data included specified, solicited symptoms, collected by diary cards during D0–6 after each vaccination; unsolicited adverse events (AEs), collected up to 30 days after each vaccination; and serious AEs (SAEs), until the end of the study. Blood samples for the evaluation of biochemical and/or hematological parameters were taken at each study visit. Food and Drug Administration toxicity grading scales for hematology and biochemistry parameters were used [18]. Symptoms were graded according to the Common Terminology Criteria for Adverse Events [19]. Adenoviral presence in whole blood was measured by DNA polymerase chain reaction at D0, D1, D3, D7, D30, D31, D33, D37, and D60. Passive RSV surveillance was performed during the study. If a participant developed respiratory symptoms at any point after Dose 1, a nasal swab was taken to test for RSV infection by polymerase chain reaction.

### Immunogenicity Assessment

Cell-mediated immune responses were evaluated at the prevaccination (D0), post-Dose 1 (D7 and D30), and post-Dose 2 (D37 and D60) time points. The frequency of interferon (IFN) γ–secreting T-cells, of anti-F immunoglobulin (Ig) G, and/or of IgA antibody-secreting B-cells were obtained by ELISpot [20].

Further exploratory tests were performed to detect vaccine-related immune responses, such as specific CD4^+^/CD8^+^ T cell determinations by ICS/FACS, identified after in vitro stimulation as CD4+/CD8+ T cells expressing interleukin 2, IFN-γ, interleukin 4, and MIP-1β.

Humoral immune responses were assessed by determination of NAb titers against RSV-A at D0, D30, and D60 by a plaque-reducing, neutralizing test, with further characterization of palivizumab-competing antibody and anti-RSV-F IgG results by enzyme-linked immunosorbent assay. Anti-ChAd155 NAb were also measured as a tertiary objective.

### Participant Selection

Participants were healthy volunteers aged between 18–45 years. Inclusion and exclusion criteria are listed in the Supplementary Methods.

### Statistical Analysis

All data were reported by group (defined by treatment administered and, for the ChAd155-RSV groups, by dose level) using descriptive statistics, regardless of enrollment step.

The sample size of 32 participants in the ChAd155-RSV-HD group was determined based on the confidence intervals (CIs) observed around a range of AE rates; that is, with an AE rate of 25%, the CI would be 11.5–43.4%.

The safety analysis was performed on the total vaccinated cohort and included all participants with at least 1 study vaccine administration documented. The according-to-protocol (ATP) cohort for immunogenicity analysis included those who received at least 1 dose of the vaccine according to protocol, and provided postvaccination immunogenicity results for at least 1 assay. Study end points are described in the Supplementary Methods.

There was no imputation of missing data.

## RESULTS

**Figure 2.**
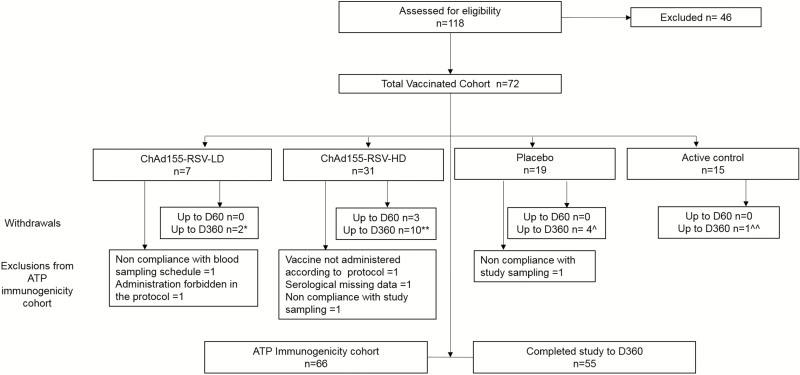
Flow diagram of the cohort. The placebo was a saline solution and the active dose was an active control. *Consent withdrawal (not due to AEs), n = 1; migrated to the study area, n = 1. **Consent withdrawal (not due to AEs), n = 2; migrated to the study area, n = 5; lost to follow-up (vaccination course completed), n = 3. ^Migrated to the study area, n = 1; lost to follow-up (vaccination course completed), n = 3. ^^Migrated to the study area, n = 1. Abbreviations: AE, adverse event; ATP, according-to-protocol; ChAd155, chimpanzee-adenovirus-155 vaccine; D, day; HD, high dose; LD, low dose; RSV, respiratory syncytial virus.

There were 72 participants who received the study vaccine, placebo, or active control as allocated (Figure 2). There were 17 participants who withdrew prior to the end of the trial. Of these, all received 2 doses of the study vaccine, apart from 2 participants in the ChAd155-RSV-HD group: 1 each withdrew after D1 and D7. There was 1 participant in the ChAd155-RSV-HD group who withdrew at D37, and all the other 14 withdrawals occurred after reaching the safety endpoint at D60. No participants withdrew due to AEs; however, 1 participant in the ChAd155-RSV-HD group declined the second dose of the vaccine due to an oral temperature of 39°C following the first dose, but continued with safety and immunogenicity tests through the duration of the study. There were 66 participants included in the ATP cohort for immunogenicity (Figure 2).

The median age at first vaccination was 28.5 years, 57% of participants were female, and participants were predominantly (89%) of Caucasian/European heritage.

### Safety

No SAEs were reported up to D60 and 2 SAEs were reported up to the study end (basal cell carcinoma in the ChAd155-RSV-LD group and acute appendicitis in the placebo group). These SAEs were considered not to be related to vaccination, and both participants recovered (Supplementary Table 1).

**Figure 3.**
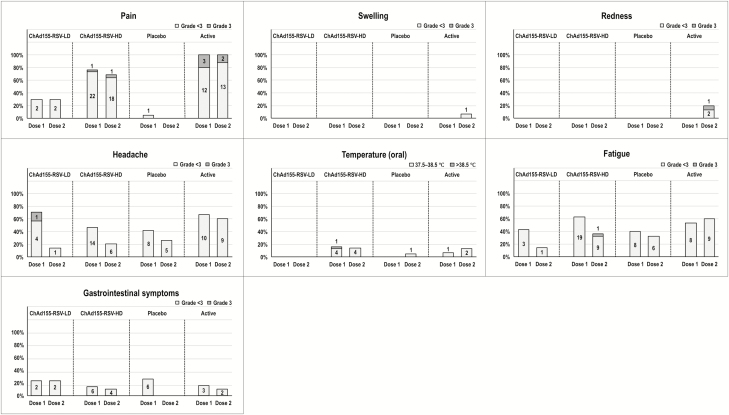
Incidence and nature of local and general solicited adverse events (AEs) during the 7-day postvaccination period following each dose. Absolute number of participants reporting AEs is indicated above each column. The placebo was a saline solution and the active dose was an active control. Abbreviations: AE, adverse event; ChAd155, chimpanzee-adenovirus-155 vaccine; D, day; HD, high dose; LD, low dose; RSV, respiratory syncytial virus.

Table 1 shows the occurrence of solicited and unsolicited symptoms within the 30-day postvaccination period; the incidence and nature of local and general solicited AEs during the 7-day postvaccination period following each dose are described in Figure 3. The most common solicited Grade 3 local AE was injection site pain, reported by 2 (7%) participants in the ChAd155-RSV-HD group and 4 (27%) participants in the active control group. The most frequently reported solicited general AEs in the ChAd155-RSV-HD arm were fatigue in 19 (63%), headache in 16 (53%), and fever in 8 (27%) participants. No Grade 3 local or general solicited AEs persisted after D6 postvaccination. There was 1 participant in the placebo arm who reported a Grade 3 oropharyngeal pain within 30 days postvaccination, which was considered to be related to the vaccination; other unsolicited symptoms within 30 days of immunization, with their causal relationships to vaccination, are shown in Supplementary Table 2.

**Table 1.** Solicited and Unsolicited Symptoms Within the 30-Day Postvaccination Period

	Study Arm	Doses, n	Any Symptom, n (%)	Grade 3, n (%)	With Causal Relationship, n (%)	Grade 3 With Causal Relationship, n (%)
*All doses*	ChAd-RSV-LD	14	12 (86)	1 (7)	10 (71)	1 (7)
	ChAd-RSV-HD	59	53 (90)	6 (10)	48 (81)	2 (3)
	Placebo	38	28 (73)	3 (8)	21 (55)	1 (3)
	Active	30	30 (100)	7 (23)	30 (100)	6 (20)
*Dose 1*	ChAd-RSV-LD	7	7 (100)	1 (14)	5 (71)	1 (14)
	ChAd-RSV-HD	31	29 (94)	4 (13)	27 (87)	1 (3)
	Placebo	19	14 (74)	3 (16)	11 (58)	1 (5)
	Active	15	15 (100)	4 (27)	15 (100)	3 (20)
*Dose 2*	ChAd-RSV-LD	7	5 (71)	0 (-)	5 (71)	0 (-)
	ChAd-RSV-HD	28	24 (86)	2 (7)	21 (75)	1 (4)
	Placebo	19	14 (74)	0 (-)	10 (53)	0 (-)
	Active	15	15 (100)	3 (20)	15 (100)	3 (20)

The placebo was a saline solution and the active dose was an active control.

Abbreviations: ChAd155, chimpanzee-adenovirus-155 vaccine; HD, high dose; LD, low dose; RSV, respiratory syncytial virus.

There was 1 subject who became pregnant approximately 33 weeks following vaccination, resulting in the birth of a healthy infant without congenital abnormalities. No clinically significant changes in biochemistry, hematology, or coagulation parameters were observed.

A total of 29 nasal swabs for the detection of RSV were performed during the study in participants with respiratory symptoms, and 2 tested positive (1 in the ChAd155-RSV-LD and 1 in the active control group). None of the 10 swabs done in the ChAd155-RSV-HD group tested positive for RSV.

### Immunogenicity

#### Cellular-mediated Immune Response

**Figure 4.**
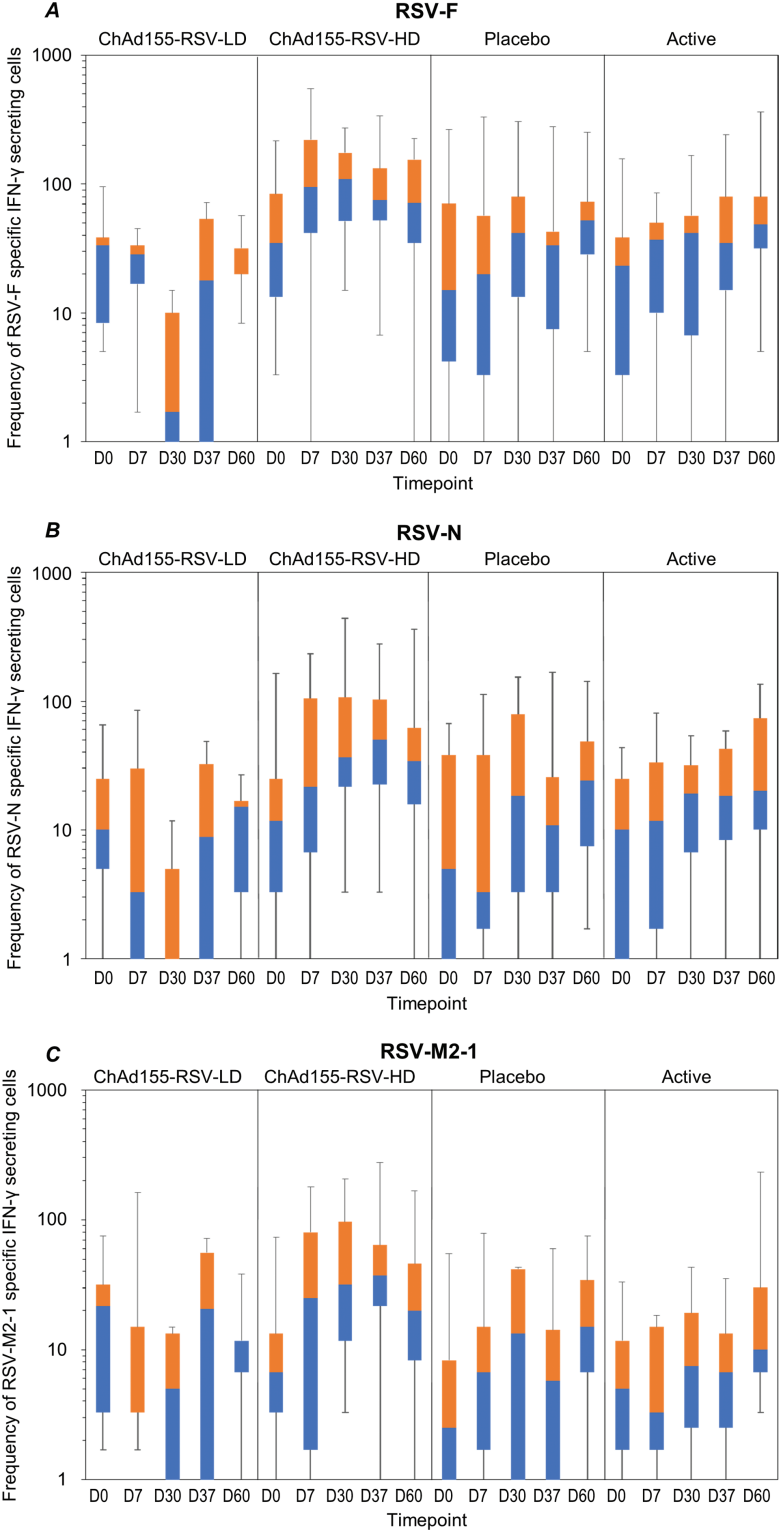
Boxplots with individual data of (*A*) RSV-F–, (*B*) RSV-N–, and (*C*) RSV-M2-1–specific IFN γ–secreting T-cells (per million of PBMCs) by ELISpot (ATP cohort for immunogenicity). The placebo was a saline solution and the active dose was an active control. Abbreviations: ATP, according-to-protocol; ChAd155, chimpanzee-adenovirus-155 vaccine; D, day; F, fusion protein; HD, high dose; IFN, interferon; LD, low dose; M2-1, antitermination protein; N, nucleocapsid protein; PMBCs, peripheral blood mononuclear cells; RSV, respiratory syncytial virus.

**Figure 5.**
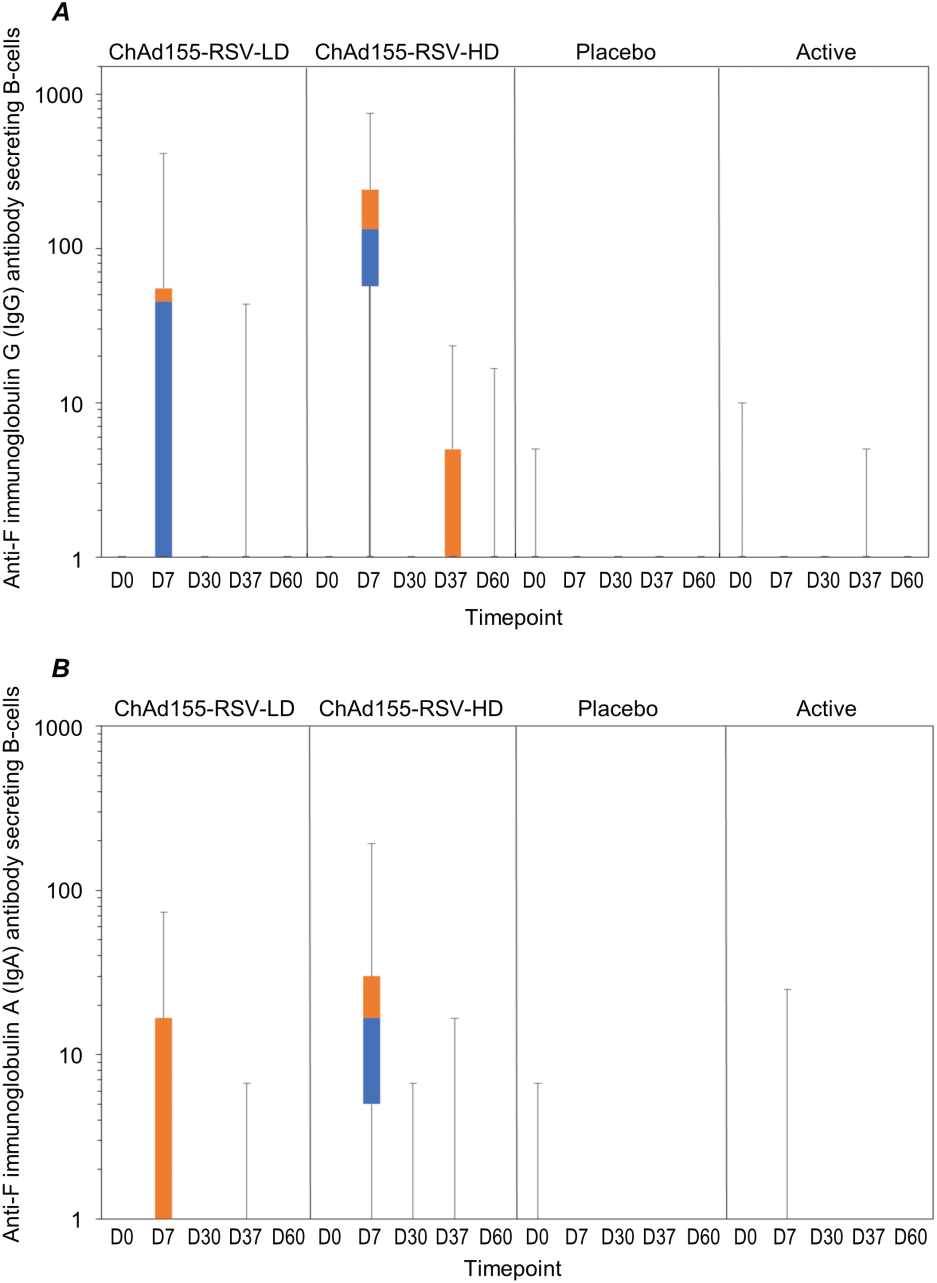
Boxplots with individual data of (A) anti-F IgG and (B) anti-F IgA antibody secreting B-cells (per million of PBMCs) by ELISpot (ATP cohort for immunogenicity). The placebo was a saline solution and the active dose was an active control. Abbreviations: anti-F, anti RSV F; ATP, according-to-protocol; ChAd155, chimpanzee-adenovirus-155 vaccine; D, day; HD, high dose; Ig, immunoglobulin; LD, low dose; PMBCs, peripheral blood mononuclear cells; RSV, respiratory syncytial virus.

In the ChAd155-RSV-HD group, the median frequency of RSV-F–specific IFN-γ–secreting T-cells (per millions of peripheral blood mononuclear cells [PBMCs]) was 35.0 (interquartile range [IQR] 13.3–83.3) at baseline, 94.2 (IQR 41.7–220.0) at D7, and 108.3 (IQR 51.7–175.0) at D30 following the first dose. No clear booster effect was observed following the second dose (Figure 4A). Similar trends were observed for RSV-N– and RSV-M2-1–specific IFN-γ–secreting T-cells (Figure 4B and 4C). No increase was observed following administration of ChAd155-RSV-LD, placebo, or active control (Figure 4). At 7 days after the first ChAd155-RSV-HD administration, the median frequency of RSV-F–specific IgG antibody–secreting B-cells (per million PBMCs) was 133.3 (IQR 56.7–240.0), and 7 days after the second dose the median was 1 (IQR 1–5) per million PBMCs (Figure 5A). Similarly, B-cells secreting anti-F IgA increased to a median value of 16.7 (IQR 5–30) at D7 after the first dose of ChAd155-RSV-HD. No further effect on this cell population was observed after the second dose (Figure 5B).

To further characterize the cellular immune response of ChAd155-RSV, the functional phenotype of vaccine-induced T cells by ICS was tested. However, the frequencies of T cells measured were not consistently sufficient to draw a robust conclusion regarding CD4+ or CD8+ T cell compositions (Supplementary Figures 1 and 2).

#### Humoral Immunity

**Figure 6.**
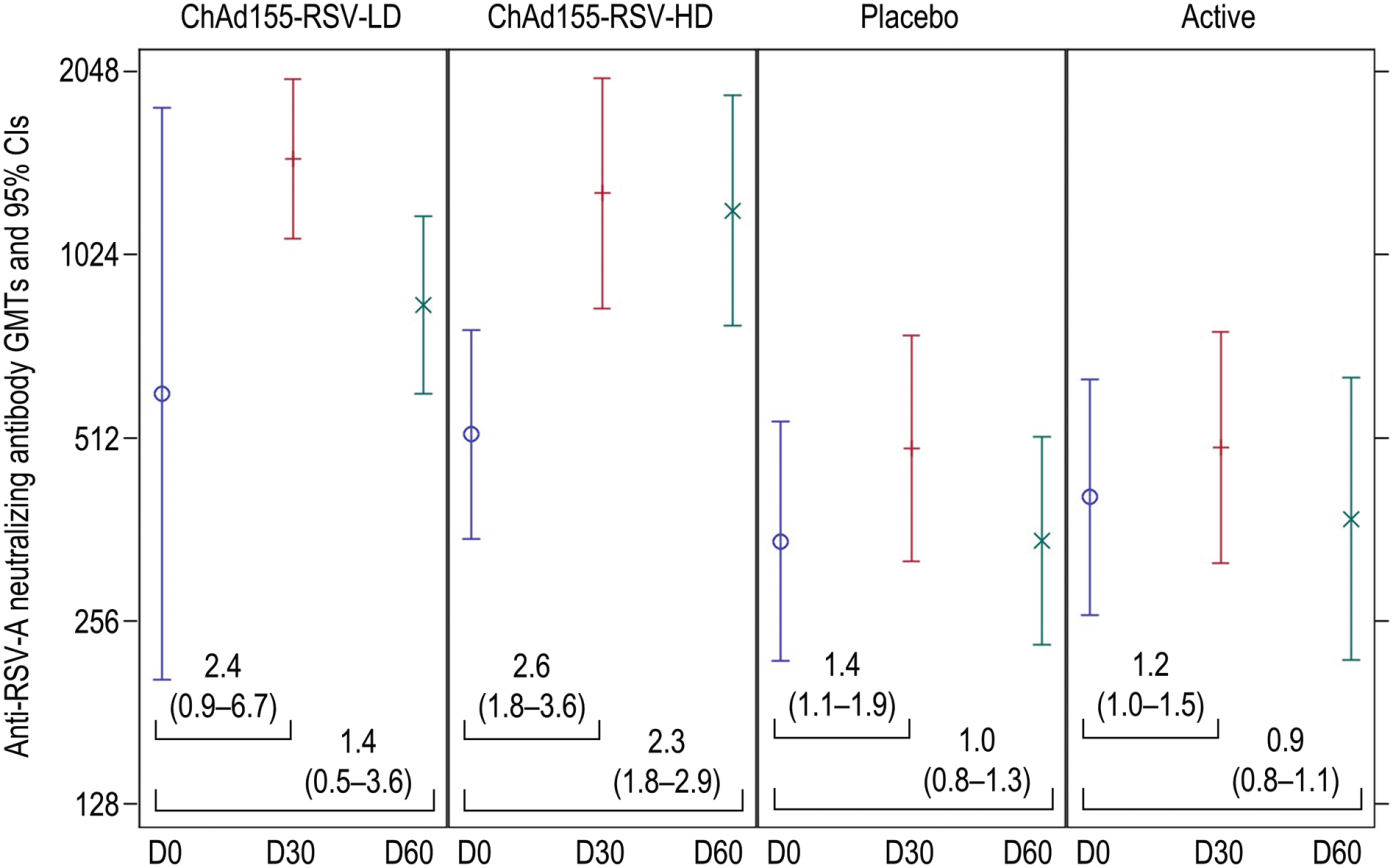
GMTs of the individual ratios of anti-RSV-A neutralizing antibody titers at each timepoint, compared to prevaccination (D0). The placebo was a saline solution and the active dose was an active control. Abbreviations: ChAd155, chimpanzee-adenovirus-155 vaccine; CI, confidence interval; D, day; GMT, geometric mean titers; HD, high dose; LD, low dose; RSV, respiratory syncytial virus.

All participants showed preexisting anti–RSV-A NAb at baseline (≥8 estimated dilution [ED] 60), with geometric mean titers (GMT) ranging from 346 (placebo) to 606 (ChAd155-RSV-LD; Figure 6; Supplementary Figure 4). At 30 days after the first dose, NAb GMTs were 2.4-fold (95% CI 0.9–6.7) and 2.6-fold (95% CI 1.8–3.6) higher than baseline for the ChAd155-RSV-LD and ChAd155-RSV-HD groups, respectively. The D30 GMT was 1474 (95% CI 1090–1993) in the ChAd155-RSV-LD group and 1293 (95% CI 835–2003) in the ChAd155-RSV-HD group (Figure 6). No further increase was observed after the second dose. No significant changes in the GMTs of RSV-A NAbs were observed in the placebo and active control groups (Figure 6). As shown in Table 2, 57.7% of participants in the ChAd155-RSV-HD group met the vaccine response definition for NAb following the first vaccine dose; no further increase in this proportion was observed after the second dose. In 3 participants (16%) in the placebo group, we observed increases in Nab, which may have been secondary to asymptomatic RSV reinfections. Vaccine responses for anti–RSV-A NAbs at each postvaccination endpoint are described in Supplementary Table 3.

**Table 2.** Vaccine Response at Each Postvaccination Timepoint

					95% CI	
Group	Postvaccination Timepoint	N	n	%	LL	UL	
ChAd155-RSV-LD	D30	5	1	20.0	.5	71.6
	D60	5	1	20.0	.5	71.6
ChAd155-RSV-HD	D30	26	15	57.7	36.9	76.6
	D60	26	15	57.7	36.9	76.6
Placebo	D30	18	3	16.7	3.6	41.4
	D60	18	1	5.6	.1	27.3
Active	D30	15	1	6.7	.2	31.9
	D60	15	2	13.3	1.7	40.5

Vaccine response was defined as: at least a 4-fold increase in prevaccination titre <7 log2; at least a 3-fold increase in prevaccination titre in [7, 8] log2; at least a 2.5-fold increase in prevaccination titre in [8–10] log2; at least a 1-fold increase in prevaccination titre >10 log2. The placebo was a saline solution and the active dose was Bexsero.Abbreviations: ChAd155, chimpanzee-adenovirus-155 vaccine; CI, confidence interval; D, day; HD, high dose; LD, low dose; LL, lower limit; N, number of participants with both pre- and postvaccination results available; n, number of participants meeting the vaccine response definition within each group; RSV, respiratory syncytial virus; UL, upper limit.

**Figure 7.**
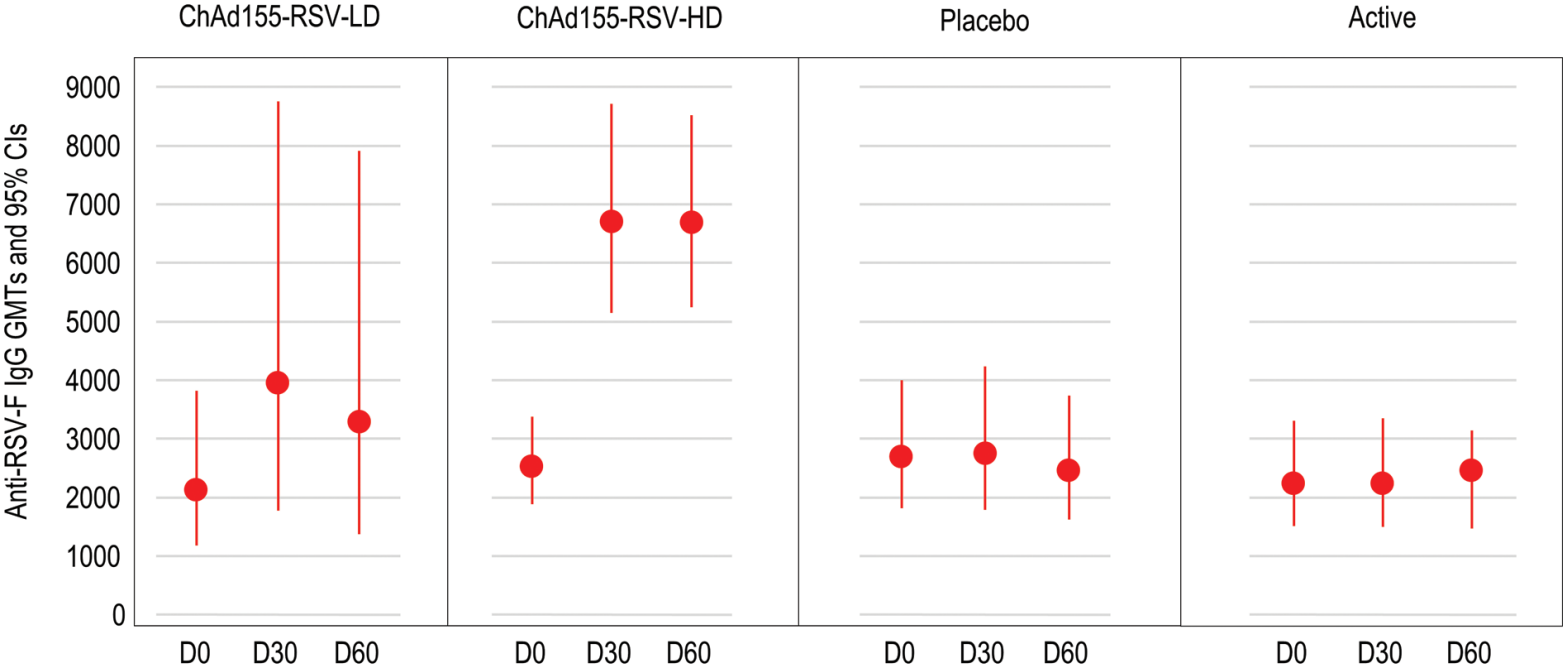
GMTs of the anti-F IgG at each timepoint. The placebo was a saline solution and the active dose was an active control. Abbreviations: anti-F, anti RSV F; ChAd155, chimpanzee-adenovirus-155 vaccine; CI, confidence interval; D, day; GMT, geometric mean titers; HD, high dose; Ig, immunoglobin; LD, low dose; RSV, respiratory syncytial virus.

All subjects had anti–RSV-F IgG values above 10 EU/ml at baseline and remained seropositive up to D60 (Figure 7); following a dose of ChAd155-RSV-HD, the anti–RSV-F IgG fold increase was 2.7, from a GMT of 2521.5 EU/ml (95% CI 1881.6–3379.1) to 6699.4 (95% CI 5148.0–8718.2). The geometric means of individual ratios of the increases in anti–RSV-F relative to anti–RSV-A NAb at D30 compared to baseline, at D60 compared to D30, and at D60 compared to baseline were 1.01, 1.17, and 1.14, respectively, in the ChAd155-RSV-HD group.

In the ChAd155-RSV-HD group, 14.3% (95% CI 4.0–32.7), 50.0% (95% CI 29.9–70.1), and 57.7% (95% CI 36.9–76.6) of subjects had palivizumab-competing antibody concentration above the lower limit of quantification at D0, D30, and D60, respectively, with geometric mean fold increases above baseline of 1.5 (95% CI 1.2–1.8) and 1.7 (95% CI 1.3–2.2) at D30 and D60, respectively.

In the ChAd155-RSV-HD group, 67.9% (95% CI 47.6–84.1) of participants had pre-vaccination (D0) ChAd155 NAb measurements ≥18 ED50, and 25.0% (95% CI 10.7–44.9) had values ≥200. At D30, the RSV-A NAb GMT was 2761 (95% CI 1387–5497) in those with ChAd155 prevaccination NAb values <18, compared to 1275 (95% CI 657–2473) and 425 (95% CI 297–609) in those with anti-ChAd155 NAb values 18–200 and >200, respectively. No trends between the levels of anti–RSV-A NAb and anti–prevaccination ChAd155 NAb titers were observed in the ChAd155-RSV-LD group (Supplementary Figure 5). Supplementary Table 4 provides a humoral immunogenicity summary.

### Detection of Chimpanzee-Adenovirus-155 Respiratory Syncytial Virus DNA by Polymerase Chain Reaction

No ChAd155-RSV adenovector DNA was detected in any of the blood samples up to 30 days after Dose 2, indicating there was no replication of the adenovirus.

### Respiratory Syncytial Virus Infections

There were 2 participants identified as having RSV infections during the study: 1 each in the ChAd155-RSV-LD and active control groups.

## DISCUSSION

The results from this first-in-human study show that immunization with ChAd155-RSV is able to generate specific cellular and humoral immune responses, is well tolerated, and has no significant safety concerns. Solicited symptoms following ChAd155-RSV administration were generally mild to moderate in severity, were transient, and were consistent with those previously reported in similar studies with adenoviral vectored vaccines [20, 21].

It has been previously reported that NAbs are insufficient to prevent reinfection with RSV, but they can reduce the risks of RSV-associated severe diseases and hospitalization [22]. Accordingly, the observed humoral immune response following the administration of ChAd155-RSV-HD is encouraging. ChAd155-RSV was able to induce more than a 2-fold increase in NAb titers, and approximately 60% of participants in the ChAd155-RSV-HD group met the predefined criteria for a vaccine response. This is despite RSV-specific serum preexisting antibodies being present in all participants, reflecting the universality of RSV infections throughout life. The evidence of high titers of preexisting antibodies may explain the lack of immune responses in the 40% of our study population.

In healthy adults protected against severe RSV respiratory disease, the majority of the NAb repertoire is directed against the prefusion conformation of the RSV-F protein [23]. A potential limitation of the study is that the conformation of the F protein encoded by the ChAd155-RSV vaccine and its expression after vaccination have not been fully determined. However, it was important to explore whether immunization with ChAd155-RSV generates functional antibodies, especially as the antibodies induced by the formalin-inactivated (FI) RSV vaccine linked to ERD were not neutralizing [24]. Reassuringly, the geometric mean ratio between the fold-rise of anti–RSV-F antibodies of IgG and the fold-rise of anti–RSV-A NAb titers was close to 1 after the first and second doses in the ChAd155-RSV-HD groups, indicating that neutralizing antibodies are proportionally induced, as compared to total F Abs [25].

The potential risk of ERD can also be minimized by the induction of a balanced Th1/Th2 immune response, given that a strongly biased Th2-like immune response to the FI-RSV vaccine was also linked to ERD upon RSV exposure [24], which is a potential strength of the adenoviral vaccines [26]. After the first dose of ChAd155-RSV-HD, a tendency towards increases in RSV-F–, -N–, and -M2-1–specific IFN-γ secreting T cells was observed, suggesting a Th1-directed immunogenicity. No increase was observed following the low dose of the study vaccine, the placebo, or the active control. While these data (along with evidence of NAb production) are in line with preclinical data showing no evidence of ERD from ChAd155-RSV immunization in mice, cotton rats, and bovine calves [27], they should be interpreted with the caveat that our study population had prior exposure to RSV, which was a significant factor that conferred protection to ERD in older infants following FI-RSV.

The anti-RSV immune responses observed following immunization with ChAd155-RSV were seen despite the presence at baseline of antibodies cross-reactive to the viral vector in some of the adult participants, which was observed to reduce the induction of anti-RSV NAb. This, combined with the preexisting immunity to RSV, may explain the diminished response to the second vaccine dose. These high rates of baseline immunity may reflect a limitation of this study; namely, that it was conducted in adults rather than the target population of infants, who were likely to have low levels of immunity to both RSV and ChAd155. RSV infection in adults can be asymptomatic, and the number of volunteers reinfected during the study may have been underestimated. However, the peak of RSV season in the United Kingdom in 2015–2016 occurred before the majority of participants were enrolled in the HD group, where no RSV reinfections were observed (Supplementary Figure 3) [28]. The safety and immune responses to 2 doses of ChAd155-RSV in 12- to 23-month-old children seropositive for RSV are currently being studied in clinical trial NCT02927873, providing a crucial step towards the evaluation of this vaccine in RSV-naive infants: the group most likely to benefit from protection.

Another limitation to the study is the high number of withdrawals (17; 24%); however, none of these were for AEs, and all but 3 provided safety data at D60 following the second vaccine dose.

In conclusion, this study demonstrates that in adults previously naturally exposed to RSV, ChAd155-RSV generates increases in specific humoral and cellular immune responses without raising significant safety concerns.

## Supplementary Data

Supplementary materials are available at *Clinical Infectious Diseases* online. Consisting of data provided by the authors to benefit the reader, the posted materials are not copyedited and are the sole responsibility of the authors, so questions or comments should be addressed to the corresponding author.

ciz653_suppl_Supplementary_Figure_1

ciz653_suppl_Supplementary_Figure_2

ciz653_suppl_Supplementary_Figure_3

ciz653_suppl_Supplementary_Figure_4

ciz653_suppl_Supplementary_Figure_5

ciz653_suppl_Supplementary_Table_1

ciz653_suppl_Supplementary_Table_2

ciz653_suppl_Supplementary_Table_3

ciz653_suppl_Supplementary_Table_4

ciz653_suppl_Supplementary_Methods

## Notes

***Author contributions***. P. K., C. d.L., P. M., L. A. F., M. P., I. D., and M. D. S. designed the study. P. C., C. J., E. S., L. S. R., P. K., C. d.L., C. H., P. M., M. J., M. P., A. G. L., I. D., and M. D. S. collected the data. P. C., C. J., E. S., L. S. R., P. K., C. d.L., C. H., M. P., A. G. L., I. D., and M. D. S. performed the study. P. C., C. J., E. S., L. S. R., P. K., C. d.L., C. H., P. M., M. J., L. A. F., M. P., A. L., A. G. L., I. D., and M. D. S. analyzed or interpreted the data. All authors had full access to the data and the corresponding author was responsible for submission of the publication. Editorial assistance and publication coordination were provided by Quentin Deraedt (Modis on behalf of GSK).

***Acknowledgments.*** The authors thank the participants who took part in the study, as well as the study co-investigators, Drs Rebecca Winslow, Devika Tharumaratnam, Malick Gibani, James Meiring; the Oxford Vaccine Group Lab staff, Sagida Bibi, Luke Blackwell, Elizabeth Jones, Susana Camara, Sonu Shrestha; the Oxford Vaccine Group research nurses; Svetlana Milca (Oxford clinical trials administrative coordinator); Margeaux Gibson (GSK local delivery lead); Sarah Wheeler (freelance clinical research associate on behalf on GSK); and Wayne Woo (GSK study statistician).

***Disclaimer.*** *Bexsero* is a trademark owned by or licensed to the GSK group of companies.

***Financial support.*** This work was supported by GlaxoSmithKline Biologicals SA.

***Potential conflicts of interest.*** P. K. received a grant from the Wellcome Trust outside the submitted work (investigator award WT109965MA). P. M., M. J., L. A. F., M. P., A. L., A. G. L., and I. D. are employees from the GSK group of companies. P. M., M. P., A. L., and I. D. own shares or stock options from GSK as part of their employee remuneration. P. C., C. J., E. S., L. S. R., C. d.L., and C. H. declare no potential conflicts of interest. M. D. S. has received grants from GSK during the conduct of the study; grants and other support from GSK; and grants from Pfizer, Medimmune, Novavax, Sanofi-Pasteur, and Johnson and Johnson, outside the submitted work. All authors have submitted the ICMJE Form for Disclosure of Potential Conflicts of Interest. Conflicts that the editors consider relevant to the content of the manuscript have been disclosed.
