# Supplementary figures and images for: First-in-Human Randomized Study to Assess the Safety and Immunogenicity of an Investigational Respiratory Syncytial Virus (RSV) Vaccine Based on Chimpanzee-Adenovirus-155 Viral Vector–Expressing RSV Fusion, Nucleocapsid, and Antitermination Viral Proteins in Healthy Adults
Source: Clin Infect Dis. 2019 Jul 24;70(10):2073–81. doi: 10.1093/cid/ciz653 (PMC7201425; doi:10.1093/cid/ciz653)

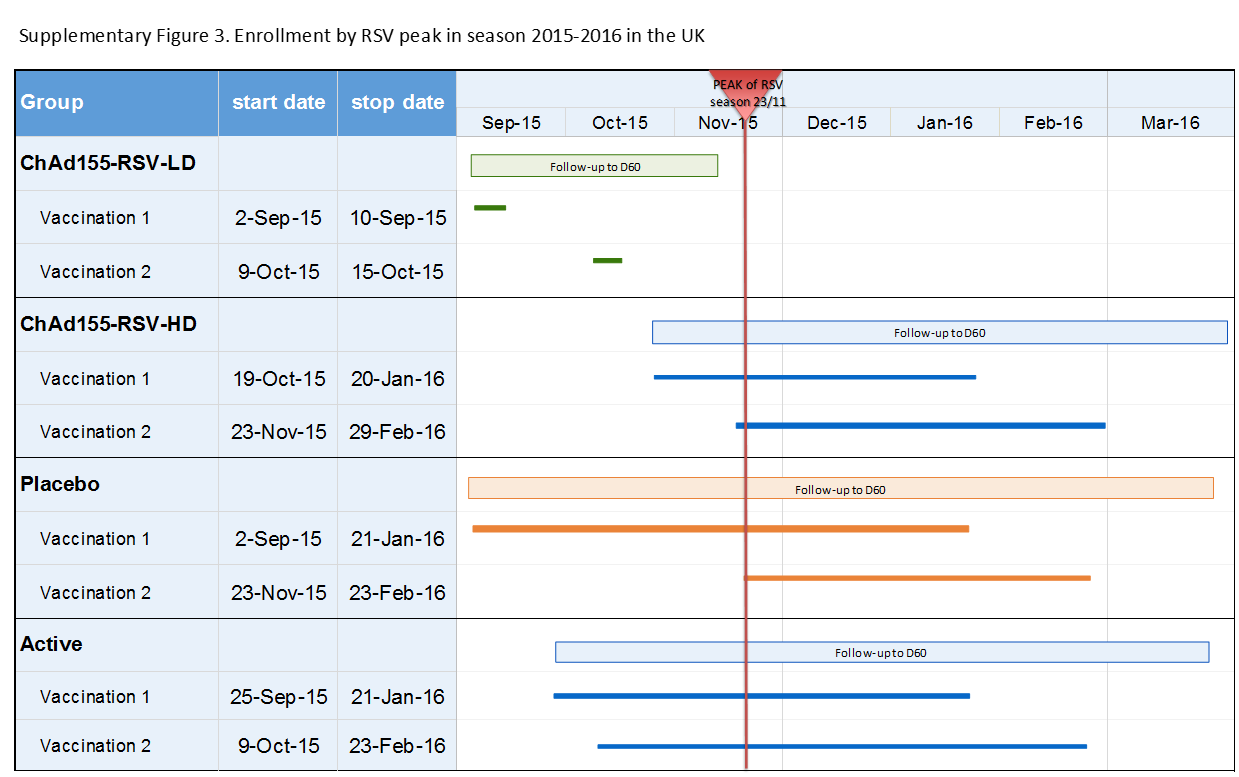

Supplement: ciz653_suppl_Supplementary_Figure_3 [file ciz653_suppl_supplementary_figure_3.png]

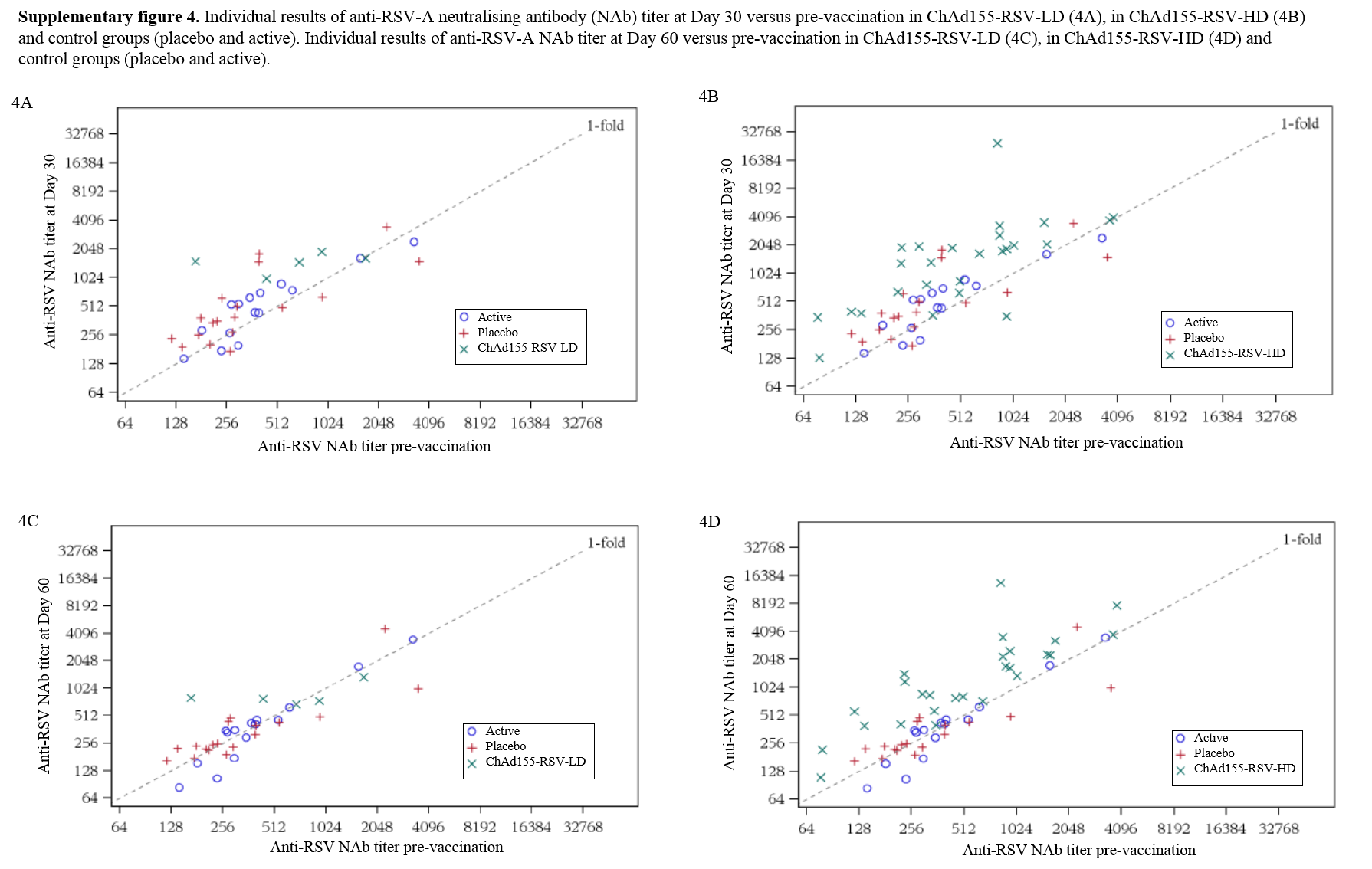

Supplement: ciz653_suppl_Supplementary_Figure_4 [file ciz653_suppl_supplementary_figure_4.png]

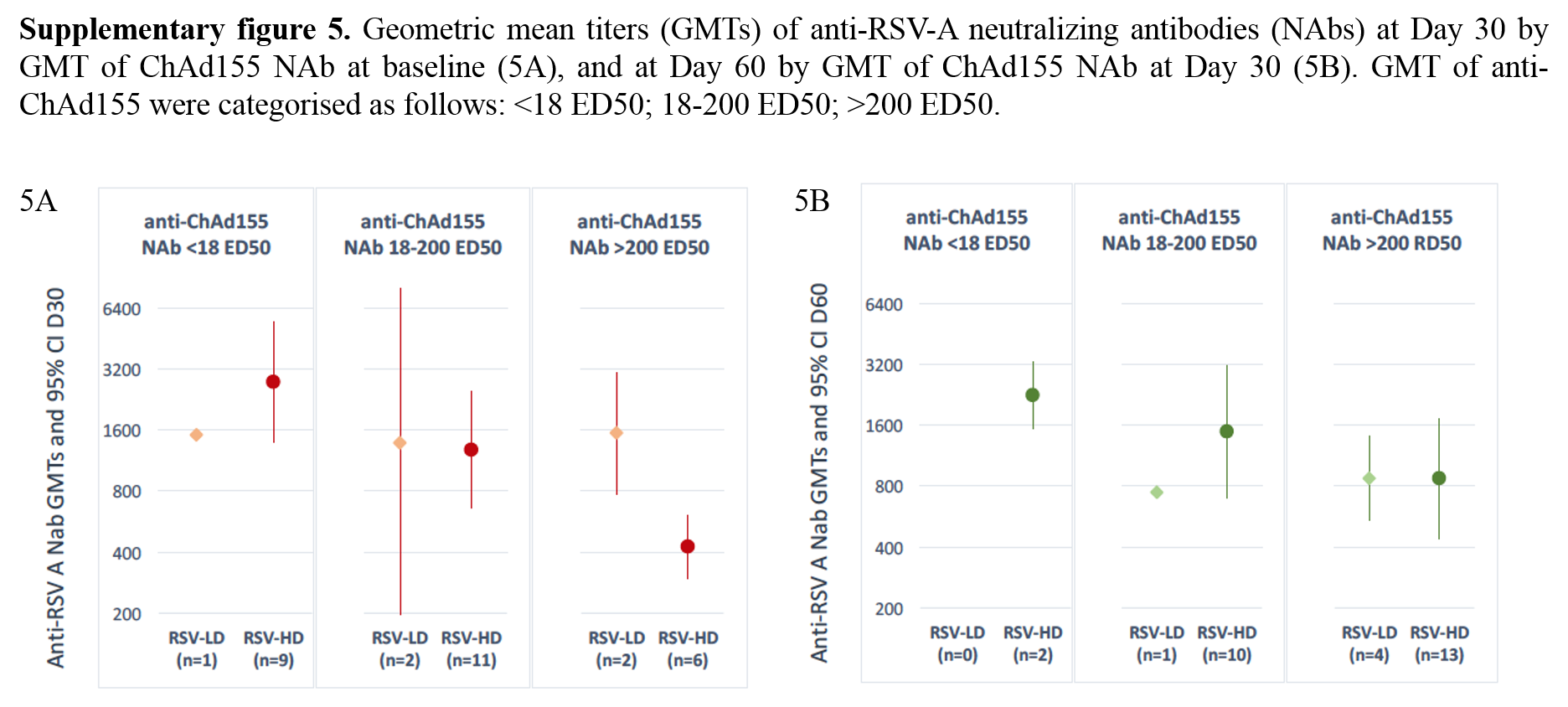

Supplement: ciz653_suppl_Supplementary_Figure_5 [file ciz653_suppl_supplementary_figure_5.png]
