# Supplementary material for: First-in-Human Randomized Study to Assess the Safety and Immunogenicity of an Investigational Respiratory Syncytial Virus (RSV) Vaccine Based on Chimpanzee-Adenovirus-155 Viral Vector–Expressing RSV Fusion, Nucleocapsid, and Antitermination Viral Proteins in Healthy Adults
Source: Clin Infect Dis. 2019 Jul 24;70(10):2073–81. doi: 10.1093/cid/ciz653 (PMC7201425; doi:10.1093/cid/ciz653)
Supplement: ciz653_suppl_Supplementary_Table_1 [file ciz653_suppl_supplementary_table_1.docx]

Supplementary table 1. Description and listing of SAEs reported up to study end (Total Vaccinated Cohort)

| **SAEs** | **Group** | **Sex** | **Race** | **Age at onset**  **(year)** | **Event** | **Day at onset** | **Duration (days)** | **Intensity** | **Causality** | **Outcome** |
| --- | --- | --- | --- | --- | --- | --- | --- | --- | --- | --- |
| 1 | ChAD155-  RSV-LD | F | White  Caucasian | 43 | Basal Cell  Carcinoma | 194 | 148 | 1 | Not  related | Recovered |
| 2 | Placebo | F | Asian | 44 | Appendicitis | 155 | 3 | 3 | Not  related | Recovered |
| Description event 1 – Basal cell carcinoma  Volunteer attended general practitioner in April 2016 for assessment of a lesion on left calf, and was referred to Dermatologist. Following assessment calf lesion was diagnosed as benign. Dermatologist noted lesion on right lower eyelid, which was subsequently diagnosed as basal cell carcinoma following histology. Participant reported lesion had been present for several years (before enrolment) and had been previously been diagnosed as benign. Subsequently underwent complete excision of basal cell carcinoma, with clear margin. Volunteer remained  asymptomatic before and after surgery. Likely cause: sun exposure. | | | | | | | | | | |
| Description event 2 – Acute appendicitis  Hospital admission for acute lower abdominal pain. Sudden onset and severe. CT consistent with acute appendicitis. The participant underwent laparoscopic appendectomy with uncomplicated recovery period. SAE was considered unrelated to IMP, given the prolonged time course since administration of vaccine/placebo. | | | | | | | | | | |
