# Supplementary material for: First-in-Human Randomized Study to Assess the Safety and Immunogenicity of an Investigational Respiratory Syncytial Virus (RSV) Vaccine Based on Chimpanzee-Adenovirus-155 Viral Vector–Expressing RSV Fusion, Nucleocapsid, and Antitermination Viral Proteins in Healthy Adults
Source: Clin Infect Dis. 2019 Jul 24;70(10):2073–81. doi: 10.1093/cid/ciz653 (PMC7201425; doi:10.1093/cid/ciz653)
Supplement: ciz653_suppl_Supplementary_Table_3 [file ciz653_suppl_supplementary_table_3.docx]

**Supplementary Table 3. Vaccine response for anti-RSV A neutralising antibody titer at each post-vaccination timepoint (ATP cohort for immunogenicity)**

|  | | |  |  | **Vaccine Response** | | | | **GMT**  **Responders** | |
| --- | --- | --- | --- | --- | --- | --- | --- | --- | --- | --- |
|  | | |  |  |  |  | **95% CI** | |  |  |
| **Test description** | **Group** | **Post- vaccination**  **timing** | **Pre- vaccination**  **category** | **N** | **n** | **%** | **LL** | **UL** | **Pre** | **Post** |
| Anti-RSV A Neutralizing Antibody | RSV-Ld | PI(D30) | 128-256 | 1 | 1 | 100 | 2.5 | 100 | 167 | 1516 |
|  |  |  | >256-1024 | 3 | 0 | 0.0 | 0.0 | 70.8 |  |  |
|  |  |  | >1024 | 1 | 0 | 0.0 | 0.0 | 97.5 |  |  |
|  |  |  | Total | 5 | 1 | 20.0 | 0.5 | 71.6 | 167 | 1516 |
|  |  | PII(D60) | 128-256 | 1 | 1 | 100 | 2.5 | 100 | 167 | 804 |
|  |  |  | >256-1024 | 3 | 0 | 0.0 | 0.0 | 70.8 |  |  |
|  |  |  | >1024 | 1 | 0 | 0.0 | 0.0 | 97.5 |  |  |
|  |  |  | Total | 5 | 1 | 20.0 | 0.5 | 71.6 | 167 | 804 |
|  | RSV-Hd | PI(D30) | <128 | 3 | 1 | 33.3 | 0.8 | 90.6 | 77 | 347 |
|  |  |  | 128-256 | 4 | 2 | 50.0 | 6.8 | 93.2 | 233 | 1589 |
|  |  |  | >256-1024 | 14 | 7 | 50.0 | 23.0 | 77.0 | 566 | 2910 |
|  |  |  | >1024 | 5 | 5 | 100 | 47.8 | 100 | 2050 | 2958 |
|  |  |  | Total | 26 | 15 | 57.7 | 36.9 | 76.6 | 676 | 2343 |
|  |  | PII(D60) | <128 | 3 | 1 | 33.3 | 0.8 | 90.6 | 121 | 563 |
|  |  |  | 128-256 | 4 | 2 | 50.0 | 6.8 | 93.2 | 233 | 1292 |
|  |  |  | >256-1024 | 13 | 6 | 46.2 | 19.2 | 74.9 | 617 | 2411 |
|  |  |  | >1024 | 6 | 6 | 100 | 54.1 | 100 | 1990 | 2963 |
|  |  |  | Total | 26 | 15 | 57.7 | 36.9 | 76.6 | 777 | 2187 |
|  | Placebo | PI(D30) | <128 | 1 | 0 | 0.0 | 0.0 | 97.5 |  |  |
|  |  |  | 128-256 | 7 | 0 | 0.0 | 0.0 | 41.0 |  |  |
|  |  |  | >256-1024 | 8 | 2 | 25.0 | 3.2 | 65.1 | 398 | 1648 |
|  |  |  | >1024 | 2 | 1 | 50.0 | 1.3 | 98.7 | 2278 | 3443 |
|  |  |  | Total | 18 | 3 | 16.7 | 3.6 | 41.4 | 712 | 2107 |
|  |  | PII(D60) | <128 | 1 | 0 | 0.0 | 0.0 | 97.5 |  |  |
|  |  |  | 128-256 | 7 | 0 | 0.0 | 0.0 | 41.0 |  |  |
|  |  |  | >256-1024 | 8 | 0 | 0.0 | 0.0 | 36.9 |  |  |
|  |  |  | >1024 | 2 | 1 | 50.0 | 1.3 | 98.7 | 2278 | 4585 |
|  |  |  | Total | 18 | 1 | 5.6 | 0.1 | 27.3 | 2278 | 4585 |
|  | Active | PI(D30) | 128-256 | 3 | 0 | 0.0 | 0.0 | 70.8 |  |  |
|  |  |  | >256-1024 | 10 | 0 | 0.0 | 0.0 | 30.8 |  |  |
|  |  |  | >1024 | 2 | 1 | 50.0 | 1.3 | 98.7 | 1591 | 1626 |
|  |  |  | Total | 15 | 1 | 6.7 | 0.2 | 31.9 | 1591 | 1626 |
|  |  | PII(D60) | 128-256 | 3 | 0 | 0.0 | 0.0 | 70.8 |  |  |
|  |  |  | >256-1024 | 10 | 0 | 0.0 | 0.0 | 30.8 |  |  |
|  |  |  | >1024 | 2 | 2 | 100 | 15.8 | 100 | 2297 | 2482 |
|  |  |  | Total | 15 | 2 | 13.3 | 1.7 | 40.5 | 2297 | 2482 |

RSV-Ld = Low dose ChAd155-RSV vaccine (5 x 10^9 vp) RSV-Hd = High dose ChAd155-RSV vaccine (5 x 10^10 vp) Placebo = Placebo

Active = Active control (Bexsero)

Total = all subjects with pre-vaccination result available Vaccine response defined as :

For subjects with pre-vaccination titer <128: antibody titer at post-vaccination ≥ 4 fold the pre-vaccination antibody titer

For subjects with pre-vaccination titer in [128-256]: antibody titer at post-vaccination ≥ 3 fold the pre-vaccination antibody titer For subjects with pre-vaccination titer in (256-1024]: antibody titer at post-vaccination ≥ 2.5 fold the pre-vaccination antibody titer

For subjects with pre-vaccination titer >1024: antibody titer at post-vaccination ≥ 1 fold the pre-vaccination antibody titer

N = number of subjects with both pre- and post-vaccination results available n/% = number/percentage of responders

95% CI = exact 95% confidence interval, LL = Lower Limit, UL = Upper Limit PI(D30)= Post-vaccination at Day 30

PII(D60)= Post-vaccination at Day 60
