# Supplementary material for: First-in-Human Randomized Study to Assess the Safety and Immunogenicity of an Investigational Respiratory Syncytial Virus (RSV) Vaccine Based on Chimpanzee-Adenovirus-155 Viral Vector–Expressing RSV Fusion, Nucleocapsid, and Antitermination Viral Proteins in Healthy Adults
Source: Clin Infect Dis. 2019 Jul 24;70(10):2073–81. doi: 10.1093/cid/ciz653 (PMC7201425; doi:10.1093/cid/ciz653)
Supplement: ciz653_suppl_Supplementary_Table_4 [file ciz653_suppl_supplementary_table_4.docx]

Supplementary table 4. Summary table of humoral immunogenicity

| Study arm | Time point | Neutralising Ab | | | PCA | | Anti-F IgG | |
| --- | --- | --- | --- | --- | --- | --- | --- | --- |
|  |  | GMT (95%CI) | GM fold increase  * (95%CI) | % vaccine response (95%CI) | GMC | GM fold increase* (95%CI) | GMC | GM fold increase* |
| ChAd155-RSV-LD | D0 | 605.9  (205.1-1789.9) |  |  | 5.9  (3.3-10.6) |  | 2123  (1778-3826.1) |  |
|  | D30 | 1474.1  (1090-1993.4) | 2.4  (0.9-6.7) | 20  (0.5-71.6) | 6.8  (3.7-12.5) | 1.2  (0.6-2.1) | 3947.1  (1778.1-8761.9) | 1.9  (0.8-4.4) |
|  | D60 | 847.4  (605.6-1185.7) | 1.4  (0.5-3.6) | 20  (0.5-71.6) | 4.8 (4.8-4.8) | 0.8  (0.5-1.4) | 3289.3  (1367.6-7911.3) | 1.5  (0.7-3.4) |
| ChAd155-RSV-HD | D0 | 519.44  (396.4-771.7) |  |  | 5.7  (4.8-6.7) |  | 2521.5  (1881.6-3379.1) |  |
|  | D30 | 1293.1  (834.8-2002.7) | 2.6  (1.8-3.6) | 57.7  (36.9-76.6) | 8.5  (6.6-11) | 1.5  (1.2-1.8) | 6699.4  (5148.0-8718.2) | 2.6  (1.9-3.5) |
|  | D60 | 1211.3  (783.6-1872.5) | 2.3  (1.8-2.9) | 57.7  (36.9-76.6) | 9.7  (7.4-12.7) | 1.7  (1.3-2.2) | 6687.7  (5245.3-8526.7) | 2.6  (2.0-3.3) |
| Placebo | D0 | 346.1  (220-544.4) |  |  | 6.4  (5.0-8.2) |  | 2696.9  (1815.3-4006.7) |  |
|  | D30 | 492.4  (321.2-754.9) | 1.4  (1.1-1.9) | 16.7  (3.6-41.4) | 6.2  (5.0-7.8) | 1  (0.7-1.3) | 2747.9 (  1782.5-4236.2) | 1.1  (0.9-1.4) |
|  | D60 | 347.3  (234.1-515.3) | 1.0  (0.8-1.3) | 5.6  (0.1-27.3) | 6.6  (5.0-8.6) | 1  (0.8-1.4) | 2460.9  (1622.4-3732.7) | 0.9  (0.8-1.1) |
| Active | D0 | 409.4  (261.8-640.2) |  |  | 6.1  (4.6-8.1) |  | 2236.7  (1509.6-3313.9) |  |
|  | D30 | 493.8  (318.7-765.3) | 1.2  (1.0-1.5) | 6.7  (0.2-31.9) | 5.9  (4.6-7.5) | 1  (0.8-1.2) | 2240.2  (1500.9-3343.5) | 1.0  (0.9-1.1) |
|  | D60 | 376.9  (220.8-643.3) | 0.9  (0.8-1.1) | 13.3  (1.7-40.5) | 6.0  (4.6-7.9) | 1  (0.7-1.3) | 2146.8  (1467.2-3141.2) | 1.0  (0.9-1.1) |

*GM of the individual ratio of concentration at each post-vaccination compared to pre-vaccination.
