# Supplementary material for: First-in-Human Randomized Study to Assess the Safety and Immunogenicity of an Investigational Respiratory Syncytial Virus (RSV) Vaccine Based on Chimpanzee-Adenovirus-155 Viral Vector–Expressing RSV Fusion, Nucleocapsid, and Antitermination Viral Proteins in Healthy Adults
Source: Clin Infect Dis. 2019 Jul 24;70(10):2073–81. doi: 10.1093/cid/ciz653 (PMC7201425; doi:10.1093/cid/ciz653)
Supplement: ciz653_suppl_Supplementary_Methods [file ciz653_suppl_supplementary_methods.docx]

**Supplementary methods**

*anti-RSV-F IgG ELISA*

# The RSV antigen was adsorbed onto a 96-well polystyrene microplate. After washing and blocking, and dilutions of serum samples, controls and standards were added to the coated microplate. After incubation, bound IgGs were detected by the addition of a secondary anti- human IgG antibody conjugated to horse-radish peroxidase (HRP). Bound antibodies were quantified by the addition of the HRP substrate, tetramethylbenzidine (TMB). A colored product proportional to the amount of anti-RSV-F protein IgG antibodies present in the serum sample is formed, and quantified by spectrophotometry. Concentrations are expressed in arbitrary ELISA Laboratory Units per milliliter (ELU/mL). The assay cut-off was set at 10 ELU/mL.

*RSV-A neutralization assay*

# Virus neutralization was performed by incubating a fixed amount of RSV A virus strain with serial dilutions of the serum sample. The serum-virus mixture was then transferred onto a monolayer of Vero cells and incubated to allow infection of the Vero cells by non-neutralized virus and the formation of plaques. After fixation, RSV-infected cells were detected using a primary antibody directed against RSV (Polyclonal anti-RSV A IgG) and a secondary antibody conjugated to HRP, allowing the visualization and counting of the plaques of plaques after coloration with TrueBlueTM peroxidase substrate. The serum neutralizing antibody titer was expressed as the estimated dilution 60 (ED60) and corresponds to the inverse of the interpolated serum dilution that yields a 60% reduction in the number of plaques compared to the virus control wells.[1-2] The assay cut-off was set at 8 ED60.

*Palivizumab-competing antibody assay*

# F protein antigen was coated onto 96-well microplates. After a washing and a blocking step, serial two-fold dilutions of serum samples, positive control serum and palivizumab antibody reference standard were added in sequence with HRP-conjugated palivizumab and incubated to allow specific binding of antibodies directed against the F protein antigens. The HRP substrate solution (TMB/H2O2) was then added and a colored product inversely proportional to the amount of palivizumab-like antibodies contained in the test serum was quantified by reading the optical densities using a spectrophotometer. Antibody concentrations are determined after interpolation from the ELISA standard curve using a four-parameter equation and are expressed as palivizumab-equivalent antibodies in microgram per milliliter (µg/mL). The assay cut-off was set at 9.60 µg/mL.

References:

1. Barbas CF 3rd, Crowe JE Jr, Cababa D, Jones TM, Zebedee SL, Murphy BR, Chanock RM, Burton DR. Human monoclonal Fab fragments derived from a combinatorial library bind to respiratory syncytial virus F glycoprotein and neutralize infectivity. Proc Natl Acad Sci USA **1992**; 89(21):10164-8.
2. Bates JT, Keefer CJ, Slaughter JC, Kulp DW, Schief WR, Crowe JE Jr. Escape from neutralization by the respiratory syncytial virus-specific neutralizing monoclonal antibody palivizumab is driven by changes in on-rate of binding to the fusion protein. Virology **2014**; 454-455:139-44
